# Effects of self-acceptance on prosocial behavior: the mediating role of self-esteem

**DOI:** 10.3389/fpsyg.2025.1643464

**Published:** 2025-11-26

**Authors:** Yuting Li, Li Guo, Guane Yang, Ying Wu

**Affiliations:** 1 School of Humanities and Social Sciences, Shanxi Medical University, Jinzhong, Shanxi, China; 2 College of Humanities and Social Sciences, Shanxi Medical University, Jinzhong, Shanxi, China; 3 Research Center for Psychological and Health Sciences, Shanxi Medical University, Taiyuan, China

**Keywords:** self-acceptance, self-esteem, prosocial behavior, longitudinal tracing, cross-lagging

## Abstract

**Objective:**

Prosocial behavior is significant for individual and social development. Although self-acceptance and self-esteem are considered important factors influencing prosocial behavior, how self-acceptance affects prosocial behavior and the role that self-esteem plays in it are unclear. Therefore, this study aimed to explore the relationships among self-acceptance, self-esteem and prosocial behavior and to verify the mediating role of self-esteem between self-acceptance and prosocial behavior.

**Methods:**

This study was divided into three stages: first, interviews were used to construct the relationships among the three variables; second, a cross-sectional survey was conducted to establish a preliminary model; and finally, a six-month follow-up study was conducted with college students at a medical school in Shanxi, where cross-lagged analyses were used to test the direct effect of self-acceptance on prosocial behavior and the mediating role of self-esteem.

**Results:**

(1) There was a significant positive correlation between self-acceptance, self-esteem and prosocial behavior; (2) self-esteem fully mediated the relationship between self-acceptance and prosocial behavior; and (3) This study validated the mediating model in which self-acceptance indirectly promotes prosocial behavior by enhancing self-esteem among medical university students, suggesting the importance of cultivating self-acceptance and self-esteem for promoting prosocial behavior in university students.

## 1 Introductory

Prosocial behavior, defined as voluntary actions that benefit others or society, is a key indicator of an individual’s social competence and moral development (Yuan et al., 2016). While existing research has established its role in maintaining positive relationships (Wang et al., 2019) enhancing psychological wellbeing (Arslan, 2021; Miles et al., 2022), and fostering social adaptation (Nelson, 2015; Yang and Kou, 2015), the psychological mechanisms that underpin these behaviors require further elucidation. A critical synthesis of this literature suggests that while the outcomes of prosociality are well-documented, less is known about its connections with fundamental aspects of the self-system, such as self-acceptance and self-esteem. This gap is particularly salient in the context of emerging adulthood. College students represent an ideal population in which to investigate these dynamics. They are situated in a critical period of self-identity formation (Xin et al., 2012; Gremmen et al., 2018) and are navigating the transition from campus to society, which involves frequent social interactions and significant adaptive challenges. This stage is characterized by both cognitive plasticity and a heightened vulnerability to psychological distress, as evidenced by recent studies documenting elevated levels of stress, anxiety, and interpersonal difficulties in this demographic (Li et al., 2022; Conley et al., 2020). The concurrent tasks of solidifying a sense of self and engaging in complex social environments make the college years a pivotal time to examine how internal self-perceptions, namely self-acceptance and self-esteem, may dynamically influence the propensity for prosocial action. Therefore, this study aims to bridge this gap by focusing specifically on the relationships between self-acceptance, self-esteem, and prosocial behavior among university students.

Self-acceptance, defined as an individual’s positive self-attitude and affirmation of self-worth despite recognizing personal limitations, serves as a crucial psychological resource for college students navigating the critical developmental task of establishing self-identity. While extensive research has documented its intrapersonal benefits, evidence regarding its interpersonal implications remains comparatively limited. Correlational studies have consistently demonstrated that self-acceptance among college students shows significant negative relationships with various psychological maladjustments while positively predicting mental health and positive development (Zheng, 2017). However, the methodological approaches in this area have been predominantly cross-sectional, leaving causal relationships underexplored. Furthermore, existing scholarship has primarily emphasized the personal adaptive functions of self-acceptance (Zhang et al., 2019), with insufficient attention to how this self-attitude might manifest in social and interpersonal domains. Prosocial behavior represents a crucial dimension of social functioning that may be significantly influenced by self-acceptance. Emerging evidence suggests a potential connection between these constructs, though the nature of this relationship requires further elaboration. For instance, a cross-sectional investigation by Guo et al. (2025) involving 1,232 college students found that those with higher self-acceptance levels reported increased engagement in prosocial acts. Similarly, another study by Chang et al. (2024) utilizing a survey method with 538 university students observed a positive correlation between these variables. Nevertheless, the existing evidence remains predominantly correlational, highlighting the need for research that can elucidate the underlying mechanisms and directional influences between self-acceptance and prosocial behavior in the college student population.

Prosocial behavior is a complex social phenomenon wherein the influence of self-acceptance may be mediated by other psychological variables, such as self-esteem, value orientation, and self-evaluation (Ma, 1995). Among these potential mediators, this study specifically focuses on self-esteem, given its well-established theoretical and empirical linkages with both self-acceptance and prosocial outcomes. Examining how self-esteem functions between these two constructs is expected to reveal a pivotal pathway from self-acceptance to prosocial action. Self-esteem refers to an individual’s overall evaluation of their self-worth, reflecting the degree to which they perceive themselves as valuable and competent (Tian and Li, 2005). As a mediating personality variable, it exerts a broad influence on cognition, motivation, emotion, and social behavior, and is closely associated with psychological wellbeing (Zheng and Lu, 2016). Eisenberg’s theoretical model of prosocial behavior posits that individuals with higher self-esteem are more likely to engage in prosocial acts, and that self-esteem level serves as a positive predictor of such behavior (Wang and Wang, 2005). Furthermore, self-acceptance has been consistently shown to be positively correlated with self-esteem (Zhan et al., 2022). Within the framework of Self-Determination Theory, self-acceptance acts as a facilitator of intrinsic motivation, aiding individuals in fulfilling their basic psychological needs (Zhang et al., 2010). When individuals are capable of self-acceptance, they are more likely to experience a sense of autonomy and competence, which in turn fosters higher self-esteem (Shi, 2001). Individuals with high self-esteem typically report a stronger sense of belonging and self-efficacy, enabling them to engage more actively in social contexts and relate more effectively to others. This propensity for active social engagement, driven by a positive self-view, translates directly into increased prosocial conduct. Empirical evidence supports this link in student populations: for instance, research indicates that self-esteem significantly predicts the tendency to engage in daily prosocial behaviors among university students (Kausar et al., 2023), and a longitudinal study found that higher baseline self-esteem predicted greater engagement in altruistic acts 1 year later (Fu et al., 2017). These findings collectively underscore the role of self-esteem as a key bridge between self-perception and social behavior. Against this backdrop, self-esteem is hypothesized to mediate the relationship between self-acceptance and prosocial behavior, which constitutes the central focus of this investigation.

In summary, previous studies have established a preliminary theoretical foundation for the relationships between self-acceptance, self-esteem, and prosocial behavior. However, the current body of evidence is constrained by several methodological limitations that warrant further investigation. Specifically, the reliance on cross-sectional designs limits causal inference about how self-acceptance influences prosocial tendencies through self-esteem. Moreover, the predominant use of self-report measures introduces potential issues of common-method bias. More importantly, there is a notable lack of longitudinal or intervention-based research that could capture the dynamic developmental relationships among these variables. To address these gaps, this study employs a mixed-methods approach combining in-depth interviews with longitudinal tracking. This design allows for a more comprehensive examination of the mediating role of self-esteem and the underlying mechanisms through which self-acceptance promotes prosocial behavior, ultimately aiming to provide empirically-supported guidance for developing effective intervention strategies.

### 1.1 Purpose of the study

The present study aims to investigate the relationship between self-acceptance and prosocial behavior among college students, with a specific focus on the mediating role of self-esteem. The research objectives are as follows:

(1) To examine the relationships among self-acceptance, self-esteem, and prosocial behavior;
(2) To test whether self-esteem mediates the link between self-acceptance and prosocial behavior;
(3) To explore the temporal and causal relationships among these variables using a cross-lagged panel design.

### 1.2 Research hypotheses

There is a two-by-two positive correlation between self-acceptance, self-esteem and prosocial behavior, and self-esteem mediates the relationship between self-acceptance and prosocial behavior.

*Hypothesis 1*: College students’ self-acceptance, self-esteem and prosocial behavior are significantly positively correlated.

*Hypothesis 2*: Self-acceptance significantly predicts self-esteem and prosocial behavior among college students.

*Hypothesis 3*: Self-esteem mediates the relationship between self-acceptance and prosocial behavior among college students.

## 2 Findings

### 2.1 Study 1: qualitative exploration through interviews on self-acceptance, self-esteem, and prosocial behavior

#### 2.1.1 Research methodology

This study employed a semi-structured interview approach to gain an in-depth understanding of the relationships among self-acceptance, self-esteem, and prosocial behavior. The qualitative methodology was selected to capture participants’ subjective perceptions and lived experiences regarding these constructs, allowing for the exploration of nuanced psychological processes that may not be fully accessible through quantitative measures alone. Participants were recruited through the researcher’s personal networks using purposive sampling. While this approach facilitated access to participants, we acknowledge its potential limitations regarding sample representativeness. To mitigate sampling bias, efforts were made to include students from diverse academic backgrounds and demographic characteristics. The selection criterion focused on educational level (university students) to ensure participants had reached a comparable level of cognitive maturity and social experience relevant to the research questions. Prior to data collection, ethical approval was obtained from the Institutional Review Board of Shanxi Medical University (Approval No: 2023SJL71). All participants received detailed information about the study’s purpose, procedures, confidentiality protections, and their rights as research participants. Written informed consent was obtained after participants had thoroughly reviewed the consent form. The interview guide covered several thematic areas, including: (1) participants’ self-perception and acceptance of personal strengths and limitations; (2) experiences and sources of self-esteem; (3) attitudes toward and engagement in prosocial behavior; and (4) perceived connections between self-acceptance, self-worth, and helping behaviors. Interviews were conducted either in person or via telephone, with each session lasting approximately 30–60 min. With participants’ permission, all interviews were audio-recorded, and the researcher took field notes to document important observations during the sessions. The audio recordings were transcribed verbatim, and the data were analyzed using thematic analysis. This involved an iterative process of reading through the transcripts, generating initial codes, and identifying emerging themes through inductive coding. The analysis aimed to identify patterns in how participants conceptualized the relationships among self-acceptance, self-esteem, and prosocial behavior.

#### 2.1.2 Research subjects

The study employed a purposive sampling method and recruited 16 college students from a medical university in Shanxi Province, China, for semi-structured interviews. The sample size was determined based on the principle of data saturation in qualitative research, whereby recruitment ceased when no new thematic information emerged in subsequent interviews. The final sample consisted of 4 college students (25%), 6 undergraduates (37.5%), and 6 postgraduates (37.5%), with an age range of 22–31 years and a male-to-female ratio of 3:13. The inclusion criteria for participants were as follows: (1) full-time university students; (2) age ≥ 18 years; and (3) voluntary participation with signed informed consent. Exclusion criteria included: (1) a history of diagnosed severe psychological disorders; and (2) previous participation in similar thematic research. Although the sample exhibited diversity in educational background, a notable gender imbalance was present, which may influence the interpretation of prosocial behavior patterns. This limitation will be addressed in the discussion section, and future studies are recommended to adopt more balanced gender sampling strategies. Detailed demographic characteristics of the interviewees are presented in Table 1, which includes comprehensive information on each participant’s identification number, gender, age, educational level, only-child status, religious affiliation, and monthly income.

**Table 1:** Basic information of the respondents.

No.	Gender	Age	Education level	Singleton condition	Religion monthly	Income
1	Female	26	Bachelor’s degree	No	No	<3,000 yuan
2	Female	23	Master’s degree and above	No	No	<3,000 yuan
3	Male	26	Bachelor’s degree	No	No	<3,000 yuan
4	Female	31	Specialized	No	No	5,001–8,000 yuan
5	Female	24	Master’s degree and above	No	No	<3,000 yuan
6	Female	26	Master’s degree and above	No	No	<3,000 yuan
7	Female	26	Specialized	No	No	5,001–8,000 yuan
8	Female	26	Bachelor’s degree	No	No	5,001–8,000 yuan
9	Male	31	Master’s degree and above	Yes	No	3,001–5,000 yuan
10	Female	23	Master’s degree and above	No	No	<3,000 yuan
11	Female	22	Bachelor’s degree	No	No	3,001–5,000 yuan
12	Female	26	Bachelor’s degree	No	No	5,001–8,000 yuan
13	Female	24	Bachelor’s degree	No	No	<3,000 yuan
14	Female	22	Specialized	No	No	<3,000 yuan
15	Female	26	Master’s degree and above	Yes	No	5,001–8,000 yuan
16	Male	27	Specialized	No	No	5,001–8,000 yuan

#### 2.1.3 Research materials

Semi structured interviews were used in this study to collect the subjects’ self-acceptance, definition of prosocial behavior, relationship between self-acceptance and self-esteem, and impact of self-acceptance on prosocial behavior. The interviews included rating scale questions, open-ended questions, and situational interview questions to gain insights into the subjects’ self-acceptance and prosocial behavior performance in specific contexts, as well as the factors that influence their self-acceptance and prosocial behavior. For example, rating questions were used to understand subjects’ overall evaluation of their own acceptance and prosocial behavioral tendencies; open-ended questions were used to explore subjects’ understanding of self-acceptance and prosocial behavior, influencing factors, and specific experiences; situational interview questions were used to simulate real-life situations and to examine the subjects’ self-talk, emotional responses, and behavioral choices in the face of a specific situation to gain a more comprehensive understanding of the relationship between self-acceptance and prosocial behavior and the role of self-esteem. Through this diversified interview design, we aim to obtain rich, in depth, and contextually relevant data to provide a solid foundation for subsequent qualitative analyses. Semi structured interviews were used in this study and were audio transcribed and checked to ensure accuracy. There were two types of coding: exploratory and hypothesis testing. (1) Exploratory coding: open and spindle coding. Open coding extracted keywords to form a first-level code; spindle coding summarized and named content common to most interviewees, quoting the original words as much as possible. (2) Hypothesis-testing coding: predetermined response types and levels, selecting codes around respondents, controlling for standardization issues, and improving validity. Two researchers coded together to reduce reliability problems. To ensure the objectivity and reliability of the coding process, two researchers independently coded a randomly selected 20% of the interview transcripts (3 out of 16). Inter-coder reliability was assessed using Cohen’s kappa coefficient. The kappa values for primary themes (e.g., self-acceptance, prosocial behavior definition, influencing factors) ranged from 0.81 to 0.93, indicating almost perfect agreement (Landis and Koch, 1977). Any discrepancies in coding were resolved through discussion until complete consensus was reached (Landis and Koch, 1977). Subsequently, the two researchers independently coded the remaining transcripts.

#### 2.1.4 Results of the interview study

(1) Self-acceptance status of the interviewees

The average self-acceptance score of the interviewees was 19.688 (SD = 4.438), and the overall self-acceptance score was 3.08 (SD = 0.854). A low self-acceptance level was classified according to the standard deviation method (according to the standard deviation method, individuals with scores between 0 and 15 can be classified as having a low self-acceptance level); medium self-acceptance scores may range from approximately 16–23; and high self-acceptance scores may range from 24 to 32, with 3 individuals with high self-acceptance levels, 3 individuals with low self-acceptance levels and 10 people. For specific details, please see Table 2.

**Table 2:** Self-acceptance scale of the respondents.

Variable	Number of people	Percentage
Low self-acceptance	3	18.75%
Self-acceptance	10	62.50%
High self-acceptance	3	18.75%

(2) The meaning of prosocial behavior

As shown in Table 3, after coding, the prosocial behaviors mentioned by the respondents can be divided into six aspects according to the number of people mentioned, from high to low: “prosocial behaviors,” “helping others,” “no request for return,” “positive attitudes,” “contribution” and “willingness to help.”

**Table 3:** Types of prosocial behavior.

Theme and keywords	Name the person	Sample quotes
P Prosocial behavior	13	
P1 Behaviors that are close to society	9	Be close to people, be altruistic and care for others
P2 Help others	8	Willing to contribute their own strength to help others and society
P3 Not expecting any reward	5	It is an act of helping others without expecting anything in return
P4 Positive attitude	3	Pro-social behavior is positive energy, a positive attitude to face people and things in society.
P5 Contribute	3	Willing to contribute their own strength to help others and society
P6 Willing help others	2	Be friendly and helpful, but not completely unselfish.

(3) Individual factors affecting prosocial behavior

Among the interviewees, 15 indicated that prosocial behavior is related to individual personality and values. Twelve of them stated that it is associated with personal values, suggesting that individuals with higher overall qualities are more likely to help others; four mentioned that it is linked to situational stress, helping others due to pressure from social situations; three noted that it is tied to social norms, as societal values influence prosocial behavior; three noted that it is related to one’s personality, with naturally outgoing individuals having a stronger inclination toward prosocial actions; and two mentioned that it is connected to the family environment and education, noting that if the family environment is harmonious, the motivation for prosocial behavior tends to be stronger. For more details, see Table 4.

**Table 4:** Individual factors influencing prosocial behavior.

Theme and keywords	Name the person	Sample quotes
S Individual factors that influence prosocial behavior	15	
S1 Personal values	12	Personal values are the decisive factor, a person’s comprehensive quality must be the most critical
S2 The stress of situations	4	I would choose to help others because of the situational pressure.
S3 Social norm	3	The values of society, situational pressures, and personal traits
S4 Nature	3	Personality is an important factor in prosocial behavior
S5 Home education	2	Family environment and education can influence a person’s prosocial behavior

(4) Impact of self-acceptance on prosocial behavior

Among the interviewees, 13 individuals indicated that self-acceptance influences prosocial behavior. Table 5 list the various factors and manifestations that the interviewees mentioned as being influenced by self-acceptance in terms of prosocial behavior.

**Table 5:** Factors and manifestations affecting prosocial behaviors induced by self-acceptance.

Theme and keywords	Name the person	Sample quotes
Influencing factors caused by self-acceptance	13	
F1 Accept yourself	9	Only after self-acceptance can individuals develop the courage to face the world. With this courage and the resultant energy, they are then empowered to help others more effectively.
F2 Self-identity	5	People with high self-acceptance are more likely to endorse what they do when helping others.
F3 Confidence and faith	4	Self-acceptance gives one the confidence and inner strength to help others.
F4 Happy	2	This will affect future actions; with self-acceptance, one will experience greater joy when helping others
F5 Powerlessness	2	For example, if someone highly accepts themselves, their confidence will increase, leading them to recognize their own positive qualities, such as kindness. This, in turn, will reinforce that quality and motivate them to help others.

Table 6 display the average scores for the two prosocial behavior scenarios, stratified by three levels of self-acceptance: low, medium, and high. Within each self-acceptance category, the mean score represents the average of the two scenario scores, with higher scores reflecting a greater propensity to engage in the described prosocial behaviors. Like Li et al. (2013), this study employed two scenario-based questions to measure prosocial behavior. The findings revealed that individuals with high self-acceptance demonstrated significantly higher scores in both scenarios than did those with medium or low self-acceptance.

**Table 6:** Mean scores for the likelihood of prosocial behavior across different conditions.

Individual level of self-acceptance	Average score	Situation 1	Situation 2
Low self-acceptance	5.73	5.67	5.79
Self-acceptance	4.58	5.40	3.75
High self-acceptance	6.18	6.00	6.35

(5) The impact of self-acceptance on self-esteem

As shown in Table 7, 16 participants mentioned that self-acceptance influenced their self-esteem. Of these, 12 reported that self-acceptance affected their approach to tasks or their self-evaluations after failure; 8 participants specifically mentioned self-esteem, indicating that self-acceptance levels influenced their self-esteem following a setback; 6 participants noted enhanced motivation, indicating that individuals with high self-acceptance became more proactive in their endeavors; and 5 participants described diminished motivation, characterized by hesitation and reluctance to move forward.

**Table 7:** Effects of self-acceptance on self-esteem-related outcomes and their manifestations.

Theme and keywords	Name the person	Sample quotes
SS Self-esteem	16	
SS1 Self-Acceptance	12	For example, the more an individual loves and accepts themselves, imperfections and all, the more confident they become. For instance, if someone experiences failure but does not attribute it to their own inadequacy and instead accepts themselves, this experience will not diminish their self-esteem.
SS2 Self-respect	8	I think if I mess up the same task, if my self-acceptance is low, my self-esteem will take a hit. Conversely, if my self-acceptance is high, it will not.
SS3 Motivation enhancement	6	After accepting oneself, self-esteem levels will improve, gradually leading to increased confidence. For example, one might become more competent in their work and more adept in social interactions.
SS4 Motivation reduction	5	Low self-acceptance can be associated with low self-esteem, leading to indecisiveness and a reluctance to take initiative.

(6) The impact of self-esteem on prosocial behavior

As shown in Table 8, 16 participants mentioned the influence of self-esteem on their prosocial behavior. Of these, 15 reported that self-esteem affected their relationships with others, primarily by fostering satisfaction with their current situation and reducing envy, thereby facilitating helping behavior. Seven participants noted that self-esteem influenced altruistic motivation, mainly by increasing the likelihood of helping others when they feel good about themselves. Four participants mentioned that self-esteem impacted their emotional state, which in turn affected prosocial behavior, primarily by making them more proactive and willing to engage in acts such as donations when in a positive mood (e.g., while shopping with friends).

**Table 8:** Correlation and manifestations of the influence of self-esteem on prosocial behavior.

Theme and keywords	Name the person	Sample quotes
E Self-esteem	16	
E1 Self-other relationship	15	Because I’m satisfied with my current situation and do not feel jealous of others, I find it easier to help them
E2 Altruistic motives	7	I’m someone who feels good about myself, so I’m naturally inclined to help others. Even when I’m going through a period of dissatisfaction with myself, I still want to help, and afterwards, I feel better about myself too.
E3 Emotional state	4	When I’m out shopping with friends and feeling happy, I’m more likely to donate money to people begging on the street than I usually would be.

#### 2.1.5 Discussion

This study, which employed semistructured interviews with 16 university students, revealed a close interplay between self-acceptance, self-esteem, and prosocial behavior. Self-acceptance, defined as the acceptance of one’s imperfections, has emerged as a multidimensional and dynamically evolving psychological process. Prosocial behavior, encompassing actions such as “engaging with society” and “helping others,” was found to be influenced by personal values and other factors. Self-acceptance appears to foster prosocial behavior by enhancing confidence, a sense of belonging, and wellbeing while simultaneously diminishing feelings of helplessness and increasing self-efficacy. Individuals with high levels of self-acceptance were more likely to engage in prosocial actions across various situations. Self-acceptance serves as the foundation for self-esteem, bolstering self-confidence and proactivity; conversely, low levels of self-acceptance can lead to diminished self-esteem. Self-esteem, in turn, influences prosocial behavior through its impact on interpersonal relationships, altruistic motivations, and emotional states. Individuals with high self-esteem are more likely to offer help, exhibit stronger altruistic motivations, and initiate prosocial acts more readily when they experience positive emotions. The rigor of this study is reflected in the meticulous development of the interview guide, ensuring its reliability and validity through multiple rounds of discussion. The interview transcripts were professionally transcribed and verified. The use of open coding and axial coding ensured objectivity in the analysis. The participants volunteered willingly, demonstrating a strong inclination toward helping others, and were selected through a purposive sampling method. Their interest in the research topic enabled them to provide rich and detailed data. This study provides an initial exploration of the intricate relationships among self-acceptance, self-esteem, and prosocial behavior, offering a theoretical foundation and empirical support for future quantitative research. It enriches the literature on these constructs and provides insights for promoting individual mental wellbeing and fostering social harmony.

### 2.2 Study two: cross-sectional survey

#### 2.2.1 Participants

A cluster sampling method was employed to recruit university students from three medical colleges located in Sichuan, Shanxi, and Qinghai Provinces. A total of 432 questionnaires were distributed, with 416 valid responses, yielding a response rate of 96.30%. The sample comprised 177 males and 239 females. Of these, 169 were only children, and 247 were non-only children. The mean age of the participants was 23.77 years (SD = 4.06). The demographic characteristics are detailed in Table 9.

**Table 9:** Demographic characteristics.

Variable	Item	Percentage (%)	Percentage (%)
Gender	Male	177	42.5
	Female	239	57.5
Only child	yes	169	40.6
	no	247	59.4

#### 2.2.2 Measurement

(1) Prosocial Tendencies Measure (PTM)

The Prosocial Tendencies Measure (PTM), developed by Carlo (Carlo and Randall, 2002), was employed in this study. This 26-item measure assesses individual differences in prosocial behavior across six distinct dimensions: public, anonymous, compliant, altruistic, emotional, and emergency. It provides a comprehensive evaluation of prosocial tendencies in various situational contexts. Responses are recorded on a 5-point Likert scale, with higher total scores indicating a greater frequency of prosocial behavior. The Cronbach’s alpha for the PTM in this study was 0.928, indicating good reliability.

(2) Self-Esteem Scale (SES)

The Self-Esteem Scale (SES) (Wu, 2009) was used to measure global self-esteem. This 10-item scale uses a 4-point response format (1 = strongly disagree, 4 = strongly agree) and includes both positively and negatively worded items to minimize response bias due to social desirability. Higher total scores reflect higher levels of self-esteem. The SES is widely used for assessing overall self-esteem. The Cronbach’s alpha for the SES in this study was 0.896, indicating good reliability.

(3) Self-Acceptance Questionnaire (SAQ)

The Self-Acceptance Questionnaire (SAQ), developed by Cong and Gao (1999), was used to assess the participants’ level of self-acceptance. This 16-item questionnaire comprises two dimensions: self-evaluation and self-acceptance. Responses are recorded on a 4-point scale, with “1” indicating “strongly agree” and “4” indicating “strongly disagree.” Higher scores indicate a higher degree of self-acceptance. The Cronbach’s alpha for the SAQ in this study was 0.929, indicating excellent reliability.

#### 2.2.3 Procedure

(1) Common method bias (CMB) check

Prior to data collection, the primary investigator explained the study’s purpose, procedures, and ethical considerations to the participants, emphasizing confidentiality and voluntary participation. To minimize common method bias, the following steps were taken. First, the purpose of the study was clearly explained before data collection, and all participants completed the questionnaires anonymously. Second, Harman’s single-factor test was employed (Zhou and Long, 2004). An exploratory factor analysis of the variables, without rotation, revealed 10 factors with eigenvalues greater than 1. The first factor explained 27.29% of the variance, which is below the critical threshold of 40%. Additionally, a common method variance test was conducted using the latent method factor approach (Williams et al., 1996). The model fit indices revealed a significant chi-square test, with both CFI and TLI failing to reach the 0.90 threshold, while RMSEA and SRMR exceeded 0.08. The suboptimal fit indices collectively indicate that common method bias is not a significant concern in this study.

(2) Data processing

During data collection, the primary investigator explained the purpose, procedures, and ethical considerations to the participants, reiterating the principles of confidentiality and voluntary participation. Common method bias was minimized through both procedural and statistical controls. The following criteria were used to identify and exclude invalid questionnaires: (1) participants checked “I disagree” on the informed consent form; (2) the average score difference between original items and semantically similar items exceeded 15 points (“Suggesting” accurately reflects that this is an inference on the basis of the data); and (3) obviously patterned responses or selecting the same option for the entire questionnaire. The data were entered and cleaned via Excel and SPSS 18.0 statistical software, and descriptive statistics were calculated.

#### 2.2.4 Correlation analysis of the variables

The means and standard deviations for each variable are presented in Table 10. The correlational analysis revealed significant associations between gender and prosocial behavior, as well as between gender and self-acceptance. Age was not correlated with prosocial behavior but was significantly associated with self-acceptance. Pairs of variables, including prosocial behavior, self-acceptance, and self-esteem, were significantly intercorrelated. After controlling for age and gender, prosocial behavior, self-esteem, and self-acceptance remained significantly correlated.

**Table 10:** Means, standard deviations, and correlation coefficients for all the variables (*N* = 416).

Variable	M	SD	Gender	Age	PTM	SA
Gender	0.57	0.495	–			
Age	23.77	4.064	–	–		
PTM	98.13	14.211	−0.159**	−0.018	–	
SA	41.236	9.633	−0.228**	0.119*	0.226**	–
SE	30.274	5.602	−0.143**	0.09	0.307**	0.790**

***p < 0.001, **p < 0.01, *p < 0.05. The same conventions apply…ents. “Female” was coded as 1, and “Male” was coded as 0; the mean represents the proportion of male students; SA refers to self-acceptance, SE refers to self-esteem, and PTM refers to prosocial behavior, the same as below.

#### 2.2.5 Mediation analysis

Taking self-acceptance as the independent variable, prosocial behavior as the dependent variable, and self-esteem as the mediator, we employed Model 4 of the PROCESS macro in SPSS, controlling for gender and age. The bias-corrected bootstrap method with 5,000 resamples was used to test the mediation effect, and the results are presented in Table 11. The regression analysis revealed a significant total effect of self-acceptance on prosocial behavior (**B** = 0.303, *p* < 0.001).

**Table 11:** Regression analysis of the mediation model of self-esteem.

Predictor variable	Model 1		Model 2		Model 3	
	B	*t*	B	*t*	B	*t*
Age	−0.162	−0.965	−0.005	−0.129	−0.157	−0.959
Gender	−3.266	−0.326	0.440	1.257	−3.663	−2.669
SA	0.303	4.17	0.465	25.681	−0.116	−1.014
SE					0.901	4.675
*R^2^*	0.653		0.626		0.113	
*F*	9.600^***^		230.01^***^		13.028^***^	

All coefficients are unstandardized (**B**). Model 1: self-acceptance predicts prosocial behavior; Model 2: self-acceptance predicts self-esteem; Model 3: self-acceptance and self-esteem jointly predict prosocial behavior.

**Figure 1:**
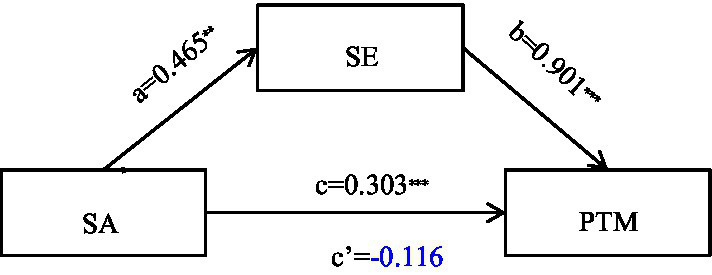
Mediation model. c = total effect, C′ = direct effect. Path coefficients are unstandardized (**B**). *p < 0.05; **p < 0.01; ***p < 0.001.

Furthermore, the bootstrap 95% confidence interval for the mediating effect was [0.232, 0.603], which did not include zero, and the mediating effect value was 0.419, accounting for 138% of the total effect. The specific results are shown in Table 12 and indicate that self-esteem fully mediates the relationship between self-acceptance and prosocial behavior. The path diagram of the mediating effect is shown in Figure 1.

**Table 12:** Total, direct, and indirect effects.

Type of the effects	Effect	SE	LLCI	ULCI
Total	0.303	0.073	0.160	0.446
Direct	−0.116	0.114	−0.341	0.109
Indirect effects	0.419	0.094	0.232	0.603

#### 2.2.6 Discussion

This study employed a cross-sectional survey design to investigate the relationships among self-acceptance, self-esteem, and prosocial behavior among university students from three provinces in China: Sichuan, Shanxi, and Qinghai. We also examined the mediating role of self-esteem in the relationship between self-acceptance and prosocial behavior. The findings provide empirical support for a better understanding of the psychological mechanisms underlying prosocial behavior in university students and offer valuable implications for promoting their mental health and social adaptation.

First, the results indicate significant positive correlations among self-acceptance, self-esteem, and prosocial behavior. These findings are generally consistent with previous research (Waterman, 1993). Individuals who practice self-acceptance tend to view themselves positively, including both their strengths and weaknesses. This positive self-perception and emotional experience contribute to the development of healthy self-esteem (Deci and Ryan, 2000). Healthy self-esteem, in turn, is associated with reduced defensiveness, increased confidence, and a more positive sense of self-worth, which may facilitate greater engagement in prosocial behavior (Twenge and Campbell, 2009). This study revealed that self-acceptance not only directly influences prosocial behavior but also indirectly affects it through self-esteem, further confirming the central role of self-acceptance in individual psychology and behavior. Second, this study revealed that self-esteem fully mediates the relationship between self-acceptance and prosocial behavior. The non-significant direct effect (**B** = −0.116, *p* > 0.05) in the presence of a significant indirect effect suggests a possible suppression effect, where self-esteem accounts for most of the variance in the relationship between self-acceptance and prosocial behavior. This study revealed that self-esteem fully mediates the relationship between self-acceptance and prosocial behavior. This finding has important theoretical implications. Self-acceptance theory posits that self-acceptance is a core component of mental wellbeing, associated with reduced psychological distress, greater life satisfaction, and more positive self-development (Twenge and Campbell, 2009). The findings of this study indicate that self-acceptance not only directly impacts individual mental health but also influences social behavior indirectly through its effect on self-esteem. Specifically, individuals with high levels of self-acceptance are more likely to develop healthy self-esteem, which, in turn, promotes a greater frequency of prosocial behavior. This finding supports the social function of self-acceptance, suggesting that it contributes not only to internal harmony within the individual but also to positive interactions between the individual and the external world. Furthermore, this study revealed significant correlations between gender and both prosocial behavior and self-acceptance, which is consistent with previous research (Eagly and Crowley, 1986). Females are generally perceived as being more inclined toward prosocial behavior than males are, which may be attributed to the societal role expectations of females and their potentially stronger empathic abilities (McMahon et al., 2006). Age was also significantly positively correlated with self-acceptance, aligning with Erikson (1968) theory of psychosocial development, which posits that early adulthood is a critical period for the development of self-identity and intimate relationships. With age, individuals’ self-perception and acceptance are hypothesized to become more mature and stable.

Additionally, the negative direct effect (though non-significant) in the presence of a positive total effect and significant indirect effect may indicate a potential suppression effect. This suggests that when self-esteem is included in the model, it accounts for the majority of the positive relationship between self-acceptance and prosocial behavior, leaving a non-significant direct path. This pattern is consistent with full mediation and highlights the central role of self-esteem in explaining how self-acceptance influences prosocial behavior.

The mediation analysis revealed that self-esteem significantly mediated the relationship between self-acceptance and prosocial behavior, but the effect size was small. This could be due to several factors: the influence of self-acceptance may be relatively indirect; cross-sectional studies are inherently limited in their ability to control for confounding variables; and self-report measures are subject to biases. Study 3 employs a longitudinal design to more precisely examine the mediating effect.

### 2.3 Study three: longitudinal study

#### 2.3.1 Participants

A cluster sampling method was used to select two classes from a medical school in Shanxi Province. These participants were measured at two time points 6 months apart. The initial measurement (T1) took place at the end of October 2024. A total of 726 students from two classes of medical postgraduates were administered the questionnaire in a group setting. Owing to 15 cases with missing or random responses, the effective number of questionnaires at T1 was 711. This sample included 202 males and 509 females, with a mean age of 23.54 ± 1.94 years.

Six months later, at the end of April of the following year, the same individuals from the same school were administered the second questionnaire. To avoid practice effects, all the items were randomly assigned in this administration. Finally, 671 participants who provided valid data at both time points were included in the paired analysis. This final sample comprised 193 males and 478 females, with a mean age of 23.48 ± 1.46 years. There was overall attrition of 40 participants between the two measurement points, resulting in an attrition rate of 5.63%.

#### 2.3.2 Measures

Self-acceptance was measured via the same scale as in Study 2. Cronbach’s alpha for the self-acceptance scale at T1 and T2 was 0.871 and 0.877, respectively. Prosocial behavior was measured via the same scale as in Study 2. Cronbach’s alpha for the prosocial tendencies scale at T1 and T2 was 0.935 and 0.932, respectively. Self-esteem was measured via the same scale as in Study 2. Cronbach’s alpha for the self-esteem scale at T1 and T2 was 0.898 and 0.881, respectively.

#### 2.3.3 Attrition analysis

To examine whether missing data at T2 could bias the results, Little’s MCAR test was conducted to analyze participant attrition, yielding a non-significant result (*p* = 0.441 > 0.05), suggesting that the missing data may be completely at random (Little, 1988), an analysis of variance (ANOVA) was conducted to compare participants who completed both measurements with those who did not. No significant differences were found between these two groups in terms of prosocial behavior or peer relationships (*p* > 0.05). Furthermore, no significant differences were observed between the attached and retained participants in terms of sex, age, or only child status (*p* > 0.05).

#### 2.3.4 Common method bias test

Since the data in this study were collected solely through self-report measures, it was necessary to test for common method bias among the variables used. This study primarily employed both procedural and statistical controls to mitigate common method variance. First, the research utilized a two-wave measurement design incorporating both simultaneous same-site assessments and asynchronous different-site measurements, which to some extent controlled and reduced common method effects. Second, Harman’s single-factor test was conducted, including all 52 items from the following measures: self-acceptance at T1, self-esteem at T1, prosocial behavior at T1, self-acceptance at T2, self-esteem at T2, and prosocial behavior at T2 (Podsakoff et al., 2003). The first measurement revealed 11 factors with eigenvalues greater than 1, with the first factor accounting for only 24.28% of the variance, which is below the critical threshold of 40%. The second measurement also revealed 11 factors with eigenvalues greater than 1, with the first factor accounting for only 24.34% of the variance, again below the 40% threshold (Hu and Bentler, 1999). Finally, a common method variance test was conducted using the latent method factor approach. The model fit indices revealed a significant chi-square test, with both CFI and TLI failing to reach the 0.90 threshold, while RMSEA and SRMR exceeded 0.08, indicating suboptimal fit across all indices (Williams et al., 1996). Therefore, it can be concluded that common method bias is not a significant concern in this study.

#### 2.3.5 Longitudinal measurement invariance testing

The model fit results for configural, metric, and scalar invariance of the research variables are presented in Table 13. The results indicate that all fit indices for the Self-Acceptance Scale and the Prosocial Behavior Scale met psychometric requirements. Based on the Satorra-Bentler scaled chi-square difference tests and changes in fit indices (ΔCFI, ΔRMSEA), both the Self-Acceptance Scale, Self-Esteem Scale, and Prosocial Behavior Scale demonstrated full scalar invariance, supporting their measurement stability across time points (Hu and Bentler, 1999).

**Table 13:** Longitudinal invariance testing of scales across two time points.

Model	*χ* ^2^	df	CFI	RMSEA	ΔCFI	ΔRMSEA
Self-acceptance						
M0,(configural)	1,132.571	206	0.882	0.082	—	—
M1 (mctric)	1,138.232	220	0.883	0.079	0.001	−0.003
M2 (scalar)	1,154.017	234	0.883	0.077	0.001	−0.002
Self-esteem						
M0 (configural)	1,732.938	54	0.762	0.215	—	—
M1 (metric)	1,742.546	62	0.762	0.201	0.000	−0.014
M2 (scalar)	1,748.801	70	0.762	0.189	0.000	−0.012
PTM						
M0 (configural)	2,389.625	568	0.891	0.069	—	—
M1 (metric)	2,419.953	588	0.890	0.068	−0.001	−0.001
M2 (scalar)	2,454.595	608	0.889	0.067	−0.001	−0.001

#### 2.3.6 Partial correlation analysis

To analyze the interrelationships among the variables, this study controlled for demographic variables such as gender, age, and only child status and examined the scores of self-acceptance, self-esteem, and prosocial behavior measured at two time points (T1 and T2) as dependent variables in a partial correlation analysis. The results, as shown in Table 14, indicate that self-acceptance at T1 was significantly positively correlated with prosocial behavior at T1 (*p* < 0.001) and self-esteem at T1 (*p* < 0.001). Furthermore, prosocial behavior at T1 was significantly positively correlated with prosocial behavior at T2 (*p* < 0.001), self-acceptance at T2 (*p* < 0.001), and self-esteem at T2 (*p* < 0.001). Self-esteem at T1 was significantly positively correlated with self-acceptance at T2 (*p* < 0.001), prosocial behavior at T2 (*p* < 0.001), and self-esteem at T2 (*p* < 0.001). Self-acceptance at T2 was significantly positively correlated with prosocial behavior at T2 (*p* < 0.001) and self-esteem at T2 (*p* < 0.001), and prosocial behavior at T2 was significantly positively correlated with self-esteem at T2 (*p* < 0.001). The results of the partial correlation analysis suggest that the data are suitable for further analysis.

**Table 14:** Partial correlation coefficients among the variables at two time points (*N* = 671).

Variable	1	2	3	4	5
1. SAT1	—				
2. PTMT1	0.193^***^	—			
3. SET1	0.71^***^	0.315^***^	—		
4. SAT2	0.733^***^	0.201^***^	0.595^***^	—	
5. PTMT2	0.163^***^	0.604^***^	0.265^***^	0.253^***^	—
6. SET2	0.594^***^	0.27^***^	0.689^***^	0.73^***^	0.319^***^

#### 2.3.7 Cross-lagged analysis of self-acceptance, self-esteem, and prosocial behavior

**Figure 2:**
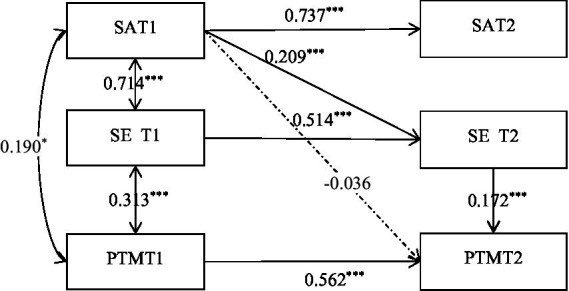
Path analysis results. The solid lines represent significant paths, whereas the dashed lines represent nonsignificant paths. *p < 0.05; **p < 0.01; ***p < 0.001.

Building upon the correlational analysis, this study employed a longitudinal design to investigate the causal relationships among self-acceptance, self-esteem, and prosocial behavior, with the theoretical model illustrated in Figure 2. Data collected from two time points (T1, T2) were analyzed using Mplus 7.3, yielding the following model fit indices: *X*^2^ = 231.154, df = 5, RMSEA = 0.000, CFI = 0.854, TLI = 0.649, SRMR = 0.066. A comprehensive examination of these fit indices indicates that the model represents a saturated model, with RMSEA = 0.000 suggesting perfect model fit and SRMR = 0.066 approaching the ideal standard, reflecting a good model-data match. Although the CFI and TLI indices did not reach optimal standards, considering the saturated nature of the model along with the excellent performance of RMSEA and SRMR, the overall model fit is deemed satisfactory. These findings provide statistical support for the research hypotheses, demonstrating that the longitudinal relationship model among self-acceptance, self-esteem, and prosocial behavior possesses explanatory power and applicability, thereby establishing a foundation for subsequent research.

In accordance with the first-order longitudinal data mediation analysis method proposed by Fang et al. (2021), a cross-temporal analysis of the mediating effects among self-acceptance, self-esteem, and prosocial behavior in university students. The bootstrap method was employed with 5,000 bootstrap samples, and significance was tested via a 95% confidence interval. According to the standardized results of the model, the path coefficient from self-acceptance at T1 (Self-Acceptance T1) to self-esteem at T2 (Self-Esteem T2) was 0.209, with a 95% confidence interval of [0.118, 0.301]. The path coefficient from self-acceptance at T1 to prosocial behavior at T2 (prosocial behavior T2) was c’ = −0.036, with a 95% confidence interval of [−0.125, 0.053]. The path coefficient from self-esteem at T2 (Self-Esteem T2) to prosocial behavior at T2 was b = 0.172, with a 95% confidence interval of [0.074, 0.271]. The longitudinal mediating effect (a*b) was 0.036, with a 95% confidence interval of [0.012, 0.068], indicating a significant longitudinal mediating effect. Furthermore, since c’ was not significant, this indicates a full mediation effect.

#### 2.3.8 Discussion

Employing a longitudinal design and cross-lagged analysis, this study investigated the relationships among self-acceptance, self-esteem, and prosocial behavior and examined the mediating role of self-esteem in the association between self-acceptance and prosocial behavior. The findings provide new insights into the developmental mechanisms underlying prosocial behavior among college students.

This study analyzed the longitudinal mediating effects among self-acceptance, self-esteem, and prosocial behavior. The results of the longitudinal mediation analysis revealed that college students’ self-esteem completely mediated the relationship between self-acceptance and prosocial behavior. The results revealed that (1) self-acceptance and self-esteem mutually predict each other, supporting the theoretical assumption that self-acceptance is a core foundation of self-esteem (Orth et al., 2012), while also validating the “sociometer theory” of self-esteem, which posits that self-esteem dynamically reflects individuals’ social adaptation status (Leary et al., 1995); (2) T2 self-esteem significantly predicts T2 prosocial behavior, and T1 prosocial behavior also positively predicts T2 self-esteem, indicating a bidirectional promotional effect between the two, which is consistent with Thomaes et al.’s (2008) view that prosocial behavior reinforces self-worth; (3) the direct effect of self-acceptance on prosocial behavior is not significant, but it indirectly influences prosocial behavior through the mediating path of self-esteem, suggesting that self-esteem is a key mechanism in their relationship. This finding deepens the understanding of the relationship between the self-system and altruistic behavior. Self-acceptance, as an individual’s fundamental recognition of their own value (Rogers, 2010), may enhance the motivation for social connection by improving self-esteem, thereby promoting prosocial behavior (Liu et al., 2024). This aligns with the theoretical framework of “self-strength” driving altruistic behavior from the perspective of positive psychology (Seligman, 2011).

This study revealed significant positive correlations among self-acceptance, self-esteem, and prosocial behavior at both time points (T1). These findings are consistent with those of previous research, suggesting that self-acceptance and self-esteem are important predictors of prosocial behavior (Eisenberg and Miller, 1987; Van der Graaff et al., 2017). Individuals with high self-acceptance tend to view themselves more positively and perceive themselves as worthy of love and respect. This positive self-perception may motivate them to help others more readily, leading to a greater display of prosocial behavior. Similarly, individuals with high self-esteem have greater confidence in their abilities and are more likely to engage in prosocial behavior.

The results of the cross-lagged analysis indicated that self-acceptance at T1 significantly and positively predicted self-esteem at T2. Furthermore, self-acceptance at T1 indirectly predicted prosocial behavior at T2 through self-esteem at T2, suggesting that self-acceptance increases prosocial behavior by increasing self-esteem levels. This finding is consistent with Bandura’s (1997) social learning theory, which posits that self-efficacy (related to self-esteem) plays a crucial role in individuals’ social behavior. Self-acceptance may promote prosocial behavior by enhancing individuals’ self-efficacy and self-confidence. From the perspective of self-determination theory, self-acceptance and self-esteem can be seen as fulfilling individuals’ needs for relatedness and competence, which in turn promotes the satisfaction of the need for autonomy, ultimately leading to intrinsic motivation-driven prosocial behavior (Wen et al., 2024).

Individuals with high self-acceptance tend to view themselves positively and believe that they are worthy of love and respect. This positive self-perception is closely related to the need for relatedness in SDT. When individuals feel accepted, loved, and respected, they are more likely to experience a sense of belonging and security, fulfilling their need for relatedness (Zhang et al., 2020). According to SDT, the fulfillment of the need for relatedness can enhance intrinsic motivation and wellbeing. When individuals feel safe and loved, they are more likely to help others out of intrinsic desire rather than external pressure or rewards. This intrinsic motivation is a key driver of prosocial behavior.

Similarly, individuals with high self-esteem have greater confidence in their abilities and are more likely to engage in prosocial behavior (Zheng and Lu, 2016). This confidence and sense of efficacy are closely related to the need for competence in SDT. The need for competence refers to the intrinsic need to feel capable and effective in facing challenges and completing tasks. Individuals with high self-esteem believe that they can effectively help others and derive a sense of achievement and satisfaction from doing so, fulfilling their need for competence (Cai, 2001). SDT posits that the fulfillment of the need for competence can enhance self-efficacy and intrinsic motivation, making individuals more willing to actively participate in prosocial behavior because they believe that they can make meaningful contributions (Fang et al., 2018). Furthermore, the satisfaction of self-acceptance and self-esteem can promote the fulfillment of the need for autonomy. When individuals feel accepted and confident, they are more likely to act in accordance with their own values and volition rather than being constrained by external pressure. The need for autonomy refers to the need to feel that one’s behavior is self-endorsed, voluntary, and aligned with one’s values and volition (Wu et al., 2018). This enhanced sense of autonomy can foster intrinsic motivation for prosocial behavior, as individuals help others out of their own choice and volition rather than out of obligation or external rewards. As mentioned above, self-acceptance and self-esteem foster intrinsic motivation by fulfilling individuals’ needs for relatedness, competence, and autonomy, thereby eliciting prosocial behavior (Zhang, 2019). This finding not only corroborates previous research but also offers a novel perspective on understanding the intrinsic motivation underlying prosocial behavior. Additionally, the sample in this study was exclusively drawn from medical institutions, where students may exhibit specific patterns in prosocial behavior due to professional characteristics (such as stronger altruistic tendencies or empathy training). Future research should validate the generalizability of this model across diverse university settings, including comprehensive and science and engineering institutions.

## 3 Contributions and limitations of the study

### 3.1 Methodological contributions

This study makes several notable methodological contributions to the literature. First, by employing a longitudinal design, it overcomes the causal inference limitations inherent in cross-sectional studies. Second, it reveals the fully mediating role of self-esteem between self-acceptance and prosocial behavior, addressing the correlational limitations of the initial studies. Third, it demonstrates the reverse effect of prosocial behavior on self-esteem, lending support to the “moral elevation hypothesis” (Martela and Ryan, 2015), which posits that altruistic behaviors enhance self-worth by fulfilling basic psychological needs (Martela and Ryan, 2015).

### 3.2 Limitations and future directions

Despite these valuable contributions, several limitations warrant acknowledgment. First, all data were collected through self-report measures, which may introduce both common method bias and social desirability bias, as participants might have exaggerated their prosocial tendencies to present themselves favorably. Although procedural and statistical controls were implemented (including Harman’s single-factor test, which showed no single factor accounted for the majority of covariance, suggesting minimal bias), these methods cannot fully eliminate potential response biases.

Second, the sample in Study 3 was drawn exclusively from a medical institution in Shanxi Province, characterized by high homogeneity in both professional focus (100% medical students) and gender distribution (78% female). This homogeneity substantially limits the generalizability of findings to other student populations and cultural contexts.

Furthermore, the longitudinal analysis relied on the standard cross-lagged panel model (CLPM), which cannot distinguish between stable between-person differences and dynamic within-person processes (Hamaker et al., 2015). Future research should employ more advanced modeling approaches, such as the random intercept cross-lagged panel model (RI-CLPM), to better disentangle these distinct effects and enhance causal inference.

Finally, while this study focused specifically on self-esteem as a mediator, it did not examine other potentially relevant psychological mechanisms. Future investigations could explore additional mediating variables, such as moral identity, self-compassion, or social connectedness, to provide a more comprehensive understanding of the relationship between self-acceptance and prosocial behavior. Taken together, these limitations provide avenues for future theoretical refinement and empirical expansion.

## 4 Conclusion

This study, conducted with a sample of medical university students, demonstrates that self-acceptance significantly predicts prosocial behavior through the mediating role of self-esteem. These findings highlight the importance of fostering self-acceptance as a means to promote prosocial engagement and offer an empirical foundation for designing targeted interventions—such as self-acceptance workshops and prosocial skill-building programs—in educational and clinical settings.

However, caution is warranted when generalizing these results to non-medical student populations or diverse cultural contexts. Future research should examine the applicability of this model across a wider range of academic environments, including disciplines such as engineering, humanities, and physical education. Additionally, further investigation is needed to elucidate the underlying mechanisms and to develop evidence-based interventions that support the positive development of university students.

## Data Availability

The original contributions presented in the study are included in the article/supplementary material, further inquiries can be directed to the corresponding author.

## References

[ref1] Arslan G. (2021). Social ostracism in school context: academic self-concept, prosocial behaviour, and adolescents’ conduct problems. Educ. Dev. Psychol. 38, 1–12. doi: 10.1080/20590776.2020.1834830

[ref2] Bandura A. (1997). Self-efficacy: the exercise of control. New York: W.H. Freeman and Company, 35, 1–610. doi: 10.5860/choice.35-1826

[ref3] Cai J.-h. (2001). Study of self-esteem among undergraduates. Chin. J. Clin. Psychol. 9, 299–301. doi: 10.3969/j.issn.1005-3611.2001.04.025

[ref4] Carlo G. Randall B. A. (2002). The development of a measure of prosocial behaviors for late adolescents. J. Youth Adolesc. 31:31. doi: 10.1023/A:1014033032440, PMID: 40797221

[ref5] Chang B. Huang J. Lin P. Huang J. (2024). Love yourself, love others more: the “altruistic” mechanism of self-compassion. Psychol. Dev. Educ. 40, 324–334. doi: 10.16187/j.cnki.issn1001-4918.2024.03.03

[ref6] Cong Z. Gao W. (1999). The development ofself-acceptance questionnaire and the testofitsreliability and validity. Chin. J. Behav. Med. Brain Sci. 8, 20–22. doi: 10.3760/cma.j.issn.1674-6554.1999.01.007

[ref7] Conley C. S. Shapiro J. B. Huguenel B. M. Kirsch A. C. (2020). Navigating the college years. Emerg. Adulthood 8:216769681879160. doi: 10.1177/2167696818791603

[ref8] Deci E. L. Ryan R. M. (2000). The ‘what’ and ‘why’ of goal pursuits: human needs and the self-determination of behavior. Psychol. Inq. 11, 227–268. doi: 10.1207/S15327965PLI1104_01

[ref9] Eagly A. H. Crowley M. (1986). Gender and helping behavior: a meta-analytic review of the social psychological literature. Psychol. Bull. 100, 283–308. doi: 10.1037/0033-2909.100.3.283, PMID: 38376350

[ref10] Eisenberg N. Miller P. A. (1987). The relation of empathy to prosocial and related behaviors. Psychol. Bull. 101, 91–119. doi: 10.1037/0033-2909.101.1.91, PMID: 3562705

[ref11] Erikson E. (1968). Identity: youth and crisis. New York: W.W. Norton & Co.

[ref12] Fang H. He B. Fu H. Zhang Y. Ma H. (2018). A review of research on the effect of need for autonomy on intrinsic motivation based on self-determination theory. Soc. Work Manag. 18, 78–83.

[ref13] Fang J. Wen Z. Qiu H. (2021). Mediation effects analysis of longitudinal data. J. Psychol. Sci. 40, 989–996. doi: 10.16719/j.cnki.1671-6981.20210431

[ref14] Fu X. Padilla-Walker L. M. Brown M. N. (2017). Longitudinal relations between adolescents' self-esteem and prosocial behavior toward strangers, friends and family. J. Adolesc. 57, 90–98. doi: 10.1016/j.adolescence.2017.04.002, PMID: 28402904

[ref15] Gremmen M. C. Berger C. Ryan A. M. Steglich C. E. G. Veenstra R. Dijkstra J. K. (2018). Adolescents’ friendships, academic achievement, and risk behaviors: same-behavior and cross-behavior selection and influence processes. Child Dev. 90, e192–e211. doi: 10.1111/cdev.13045, PMID: 29450883

[ref16] Guo L. Niu Y. Li X. Li Y. Xue Z. Yang G. (2025). Sense of meaning in life, self-acceptance, and prosocial behavior: an application of network analysis methods. Front. Psychol. 16:1533687. doi: 10.3389/fpsyg.2025.1533687, PMID: 40259999 PMC12010770

[ref17] Hamaker E. L. Kuipers R. M. Grasman R. P. (2015). A critique of the cross-lagged panel model. Psychol. Methods 20, 102–116. doi: 10.1037/a0038889, PMID: 25822208

[ref18] Hu L. Bentler P. M. (1999). Cutoff criteria for fit indexes in covariance structure analysis: conventional criteria versus new alternatives. Struct. Equ. Modeling 6, 1–55. doi: 10.1080/10705519909540118

[ref19] Kausar A. Ali N. Ismail S. (2023). Emotional expressivity as a moderator between self-esteem and prosocial behavior among undergraduate university students in Pakistan. Nurture 17:449. doi: 10.55951/nurture.v17i4.449

[ref20] Landis R. J. Koch G. G. (1977). The measurement of observer agreement for categorical data. Biometrics 33, 159–174. doi: 10.2307/2529310, PMID: 843571

[ref21] Leary M. R. Tambor E. S. Terdal S. K. Downs D. L. (1995). Self-esteem as an interpersonal monitor: the sociometer hypothesis. J. Pers. Soc. Psychol. 68, 518–530. doi: 10.1037/0022-3514.68.3.518

[ref22] Li W. Zhao Z. Chen D. Peng Y. Lu Z. (2022). Prevalence and associated factors of depression and anxiety symptoms among college students: a systematic review and meta-analysis. J. Child Psychol. Psychiatry 63, 1222–1230. doi: 10.1111/jcpp.13606, PMID: 35297041

[ref9001] Li G. Zhou H. Ding R. (2013). The effects of moral self‑regulation on prosocial behavior and rule infraction. Acta Psychologica Sinica, 45, 78–85. doi: 10.3724/SP.J.1041.2013.00672

[ref23] Little R. J. A. (1988). A test of missing completely at random for multivariate data with missing values. J. Am. Stat. Assoc. 83, 1198–1202. doi: 10.1080/01621459.1988.10478722

[ref24] Liu X. Wang C. Wang D. Liu W. (2024). School acceptance and support moderate the relationship between parental divorce and adolescents’ prosocial behavior: a longitudinal study. Appl. Res. Qual. Life 19, 905–919. doi: 10.1007/s11482-024-10275-3

[ref25] Ma X. (1995). Experimenting with the effects of self-focus on pro-social behaviour. J. Kunming Junior Normal College 26, 74–80.

[ref26] Martela F. Ryan R. M. (2015). The benefits of benevolence: basic psychological needs, beneficence, and the enhancement of well-being. J. Pers. 84, 750–764. doi: 10.1111/jopy.12215, PMID: 26249135

[ref27] McMahon S. D. Wernsman J. Parnes A. L. (2006). Understanding prosocial behavior: the impact of empathy and gender among African American adolescents. J. Adolesc. Health 39, 135–137. doi: 10.1016/j.jadohealth.2005.10.008, PMID: 16781977

[ref29] Miles A. Andiappan M. Upenieks L. Orfanidis C. (2022). Using prosocial behavior to safeguard mental health and foster emotional well-being during the Covid-19 pandemic: a registered report of a randomized trial. PLoS One 17:e0272152. doi: 10.1371/journal.pone.0272152, PMID: 35901118 PMC9333215

[ref30] Nelson S. K. (2015). The effects of prosocial and self-focused behaviors on psychological flourishing. Riverside: University of California. 10.1037/emo0000178 27100366

[ref31] Orth U. Robins R. W. Widaman K. F. (2012). Life-span development of self-esteem and its effects on important life outcomes. J. Pers. Soc. Psychol. 102, 1271–1288. doi: 10.1037/a0025558, PMID: 21942279

[ref32] Podsakoff P. M. MacKenzie S. B. Lee J.-Y. Podsakoff N. P. (2003). Common method biases in behavioral research: a critical review of the literature and recommended remedies. J. Appl. Psychol. 88, 879–903. doi: 10.1037/0021-9010.88.5.879, PMID: 14516251

[ref33] Rogers C. (2010). A theory of therapy, personality, and interpersonal relationships, as developed in the client-centered framework. New York: McGraw-Hill.

[ref34] Seligman M. (2011). Flourish: a visionary new understanding of happiness and well-being. Choice Rev. Online 48, 48–7217. doi: 10.5860/choice.48-7217

[ref35] Shi S. (2001). A psychological survey and analysis of self-esteem and self-acceptance among university students. J. Yichun Univ., 2, 97–98.

[ref36] Thomaes S. Bushman B. J. Stegge H. Olthof T. (2008). Trumping shame by blasts of noise: narcissism, self-esteem, shame, and aggression in young adolescents. Child Dev. 79, 1792–1801. doi: 10.1111/j.1467-8624.2008.01226.x, PMID: 19037950

[ref37] Tian L. Li S. (2005). Differentiating and analyzing the concept of self-esteem. Psychol. Explor. 25, 26–40. doi: 10.3969/j.issn.1003-5184.2005.02.006

[ref38] Twenge J. M. Campbell W. K. (2009). The narcissism epidemic: living in the age of entitlement. New York, USA: Simon and Schuster.

[ref39] Van der Graaff J. Carlo G. Crocetti E. Koot H. M. Branje S. (2017). Prosocial behavior in adolescence: gender differences in development and links with empathy. J. Youth Adolesc. 47, 1086–1099. doi: 10.1007/s10964-017-0786-1, PMID: 29185207 PMC5878203

[ref40] Wang L. Wang T. (2005). Research on pro-social behaviour of adolescents. Contemp. Youth Res. 11, 51–53. doi: 10.3969/j.issn.1006-1789.2005.11.009

[ref41] Wang M. Wang J. Deng X. Chen W. (2019). Why are empathic children more liked by peers? The mediating roles of prosocial and aggressive behaviors. Pers. Individ. Differ. 144, 19–23. doi: 10.1016/j.paid.2019.02.029

[ref42] Waterman A. S. (1993). Two conceptions of happiness: contrasts of personal expressiveness (eudaimonia) and hedonic enjoyment. J. Pers. Soc. Psychol. 64, 678–691. doi: 10.1037/0022-3514.64.4.678

[ref43] Wen R. Chen X. Tang Z. Chen C. (2024). The effects of social exclusion on the pro-social behavior of social workers: the mediating role of self-esteem. Adv. Psychol. 14, 178–188. doi: 10.12677/ap.2024.146396

[ref44] Williams L. J. Gavin M. B. Williams M. L. (1996). Measurement and nonmeasurement processes with negative affectivity and employee attitudes. J. Appl. Psychol. 81, 88–101. doi: 10.1037/0021-9010.81.1.88

[ref45] Wu Z. (2009). Quality control in the use of psychometric scales. Chin. Ment. Health J. 23, 837–839.

[ref46] Wu C. Rong S. Zhu F. Kan Y. Guo Y. (2018). Basic psychological need and its satisfaction. Adv. Psychol. Sci. 26, 1063–1073. doi: 10.3724/SP.J.1042.2018.01063

[ref47] Xin Z. He L. Zhang M. (2012). Changes in college students’ mental health: a cross-temporal meta-analysis. Acta Psychol. Sin. 44, 664–679. doi: 10.3724/Sp.J.1041.2012.00664

[ref48] Yang Y. Kou Y. (2015). Individuals’ well-being in prosocial interaction: the role of autonomy. Adv. Psychol. Sci. 23:1226. doi: 10.3724/SP.J.1042.2015.01226

[ref49] Yuan M. Zhang M. Kou Y. (2016). Prosocial reputation and prosocial behavior. Adv. Psychol. Sci. 24, 1655–1662. doi: 10.3724/Sp.J.1042.2016.01655

[ref50] Zhan S. Chen X. Luo F. Yang Y. Yang Z. (2022). Emotional maltreatment and social anxiety in rural college studentswith left-behind experience: the mediation effect of self-esteem and self-acceptance. Chin. J. Clin. Psychol. 30, 630–634+639. doi: 10.16128/j.cnki.1005-3611.2022.03.026

[ref51] Zhang A. (2019). Research on the psychological mechanism of ‘goodness will have a good reward’—book review of the influence of prosocial behavior on happiness and its psychological mechanism. Adv. Psychol. 9, 2042–2047. doi: 10.12677/AP.2019.912245

[ref52] Zhang J. W. Chen S. Tomova Shakur T. K. (2019). From me to you: self-compassion predicts acceptance of own and others’ imperfections. Personal. Soc. Psychol. Bull. 46, 228–242. doi: 10.1177/0146167219853846, PMID: 31185807

[ref53] Zhang X. Peng T. Hong Y. Luo J. (2020). A study of the relationship between college students’ belief in a just world, self-esteem and self-acceptance. J. Xinyang Normal Univ. (Philos. Soc. Sci. Ed.) 40, 82–87. doi: 10.3969/j.issn.1003-0964.2020.02.014

[ref54] Zhang Y. Wang Y. Fu W. Yang S. (2010). A study on the correlation between self-acceptance and achievement motivation among college students. J. North China Univ. Sci. Technol. (Health Sci. Ed.) 12, 140–141. doi: 10.3969/j.issn.1008-6633.2010.01.123

[ref55] Zheng H. (2017). A review of self-acceptance research. Modern Commun. 2017, 25–26.

[ref56] Zheng Q. Lu n. (2016). Self-esteem as mediator between parental company and pro-social behavior of 5 to 6 years old children. Chin. J. Sch. Health 37, 71–73. doi: 10.16835/j.cnki.1000-9817.2016.01.021

[ref57] Zhou H. Long L. (2004). Statistical remedies for common method biases. Adv. Psychol. Sci. 12, 942–950. doi: 10.3969/j.issn.1671-3710.2004.06.018

