# Correction: Effects of self-acceptance on prosocial behavior: the mediating role of self-esteem

**DOI:** 10.3389/fpsyg.2025.1765707

**Published:** 2026-01-29

**Authors:** Yuting Li, Li Guo, Guane Yang, Ying Wu

**Affiliations:** 1 School of Humanities and Social Sciences, Shanxi Medical University, Jinzhong, Shanxi, China; 2 College of Pharmacy, Shanxi Medical University, Jinzhong, China; 3 Research Center for Psychological and Health Sciences, Shanxi Medical University, Taiyuan, China

**Keywords:** self-acceptance, self-esteem, prosocial behavior, longitudinal tracing, cross-lagging

Author Guane Yang was erroneously spelled as Gaune Yang.

Affiliation 1 “School of Humanities and Social Sciences, Shanxi Medical University, Jinzhong, Shanxi, China” and Affiliation 2 “College of Humanities and Social Sciences, Shanxi Medical University, Jinzhong, Shanxi, China” in the published article were incorrectly presented due to inconsistent use of English translations for the same school. The correct author list with unified affiliations appears below.

Yuting Li^1^, Li Guo^1^, Guane Yang^1,2^ Ying Wu^1,3^

^1^School of Humanities and Social Sciences, Shanxi Medical University, Jinzhong, Shanxi, China

^2^College of Pharmacy, Shanxi Medical University, Jinzhong, China

^3^Research Center for Psychological and Health Sciences, Shanxi Medical University, Taiyuan, China

The original version of this article has been updated.

